# A New Criterion for Exponential Stability of a Class of Hopfield Neural Network with Time-Varying Delay Based on Gronwall's Inequality

**DOI:** 10.1155/2021/4713450

**Published:** 2021-09-13

**Authors:** Faming Guo, Ricai Luo, Xiaolan Qin, Yunfei Yi

**Affiliations:** ^1^Department of Mathematics and Physics, Hechi University, Yizhou, China; ^2^Institute of Mathematical Sciences, Guizhou Normal University, Guizhou, China; ^3^Department of Computer and Information Engineering, Hechi University, Yizhou, China

## Abstract

In this paper, we study the problem of exponential stability for the Hopfield neural network with time-varying delays. Different from the existing results, we establish new stability criteria by employing the method of variation of constants and Gronwall's integral inequality. Finally, we give several examples to show the effectiveness and applicability of the obtained criterion.

## 1. Introduction

Since Hopfield [1] proposed the Hopfield neural network named after him in 1984, these types of artificial neural networks have been widely applied in many aspects, such as combinative optimization [2–4], image processing [5, 6], pattern recognition [7], signal processing [8], communication technology [9], and so on. The Hopfield neural network has been extensively investigated in the past decades [10–26]. In the practical application of neural networks, because of the time delay of information transmission between two neurons and the influence of hardware, such as the limited speed of switch, the phenomenon of time delay is inevitable. Therefore, the introduction of a time delay in the study of neural networks has widely been of concern [15–23]. Because the number of hidden layers and the initial value of connection weights of the neural network are random, the stability of the system is not being guaranteed. If the control system is unstable, the convergence of the network will lose its foundation. Therefore, stability is a very important property for neural networks. In the study of the stability of Hopfield neural networks, researchers usually construct Lyapunov functional and combine with linear matrix inequality or integral inequality to analyze the stability of the system. It is no doubt that Lyapunov's method is a powerful tool in the study of the stability of differential equations, but how to construct an appropriate Lyapunov functional is the key to solve these problems. In addition, constructing different Lyapunov functions for the same system will lead to different stability ranges, which is also an uncertain problem. Besides, the operation of the linear matrix inequality is very complicated. Zhang et al. proposed a method based on weight delay to study the stability of a class of recurrent neural networks with time-varying delays [25]. They obtained a new delay-dependent stability criterion for neural networks with time-varying delays by constructing a Lyapunov–Krasovskii functional and using Jensen's integral inequality. However, the results obtained by the authors are complicated. To describe the complexity of these results, we give another specific example. Wang et al. [27] studied the delay-dependent stability of a class of generalized continuous neural networks with time-varying delays in system (1) (for the meaning of parameters in the formula, please refer to article [27]):

(1) $$\begin{array}{c} \dot{u}\left(t\right)=-Au\left(t\right)+B\hat{f}\left(u\left(t\right)\right)+C\hat{f}\left(u\left(t-\tau \left(t\right)\right)\right)+\hat{I}. \end{array}$$

They shift the equilibrium point **u**^*∗*^ of system (1) to the origin by the transformation **x**(*t*)=**u**(*t*) − **u**^*∗*^ and obtain

(2) $$\begin{array}{c} \dot{x}\left(t\right)=-Ax\left(t\right)+Bf\left(x\left(t\right)\right)+Cf\left(x\left(t-\tau \left(t\right)\right)\right). \end{array}$$

They constructed a new Lyapunov–Krasovskii functional and then used Jensen's integral inequality to obtain the following criterion for system (2). The origin of system (2) is globally asymptotically stable, if for given diagonal matrices Δ_1_ and Δ_2_ and positive scalars *τ*_*m*_, *τ*_*M*_, *ρ*_*m*_, *ρ*_*M*_, *β*_*k*_, *γ*_*j*_, *n*_1_, and *n*_2_, there exist symmetric definite matrices *P* > 0, *W*_*s*_ > 0, *S*_*s*_ > 0, *Q*_*k*_ > 0, and *R*_*j*_ > 0, positive definite diagonal matrices *V* > 0, *U* > 0, Λ_1_ > 0, and Λ_2_ > 0, and matrices *G*_*r*_ and *J* such that the following inequalities hold, *s*=1,2,3:

(3) $$\begin{array}{c} \Omega -\Xi_{1}-\Xi_{2}<0,\\ \left[\begin{array}{cc} S_{3} & J\\ {}* & S_{3} \end{array}\right]\geq 0,\;\max\beta_{k},\gamma_{i}\rho_{M}<1,\\ \left[\begin{array}{cc} S_{2} & G_{r}\\ {}* & S_{2} \end{array}\right]\geq 0,\;r=1,\ldots ,\frac{\left(n_{1}+n_{2}\right)\left(n_{1}+n_{2}-1\right)}{2}. \end{array}$$

These symbols are defined in [27].

There are some problems with this result:

- Do the matrices *P*, *W*_*s*_, *S*_*s*_, *Q*_*k*_, and *R*_*j*_ exist?
- For such complex matrix inequalities, how does one ensure the existence of the unknown matrices?
- If they exist, how are they represented?

If one does not solve these problems, the stability of the original equation remains unsolved. In fact, the stability depends only on the coefficient matrices of the system, not on the existence of those unknown matrices.

We have also paid attention to some recent research results [28–30]. Their conclusions are also based on the creation of the Lyapunov–Krasovskii functional. They all assume that some unknown matrices satisfying some matrix inequalities make the system stable, and it is unknown whether these unknown matrices exist.

To solve this problem, in this paper, we will use the technique of integral inequality to construct a new stability criterion, which is only related to the coefficient matrix and independent of those unknown matrices.

Gronwall's integral inequality plays an important role in the qualitative theory of differential equations. Many researchers extended it and used it to solve numerous problems [31–36]. However, it is rare to study the stability of a neural network system. In this paper, we use Gronwall's inequality to avoid the above problems and obtain new criteria for the exponential stability of a class of Hopfield neural network with a time-varying delay. Similar to the model studied by Wang et al. [27], we consider the following system:

(4) $$\begin{array}{c} \left\{\begin{array}{c} \dot{x}\left(t\right)=-Ax\left(t\right)+Bg\left(x\left(t\right)\right)+Cg\left(x\left(t-\tau \left(t\right)\right)\right)+U,\\ x\left(t\right)=\kappa \left(t\right),\;\forall t\in \left[-\tau ,0\right], \end{array}\right) \end{array}$$

where **x**(*t*)=(*x*_1_(*t*),*x*_2_(*t*),…,*x*_*n*_(*t*))^*T*^ denotes the neuron state vector, **g**(**x**(*t*))=(*g*_1_(*x*_1_(*t*)), *g*_2_(*x*_2_(*t*)),…,*g*_*n*_(*x*_*n*_(*t*)))^*T*^is the activation function, and **g**(**x**(*t* − *τ*(*t*))) is the time-delay term. **A**={diag *a*_1_, *a*_2_,…, *a*_*n*_}(*a*_*i*_ > 0, *i*=1,2,…, *n*), **B**=(*b*_*ij*_)_*n*×*n*_, and **C**=(*c*_*ij*_)_*n*×*n*_ are the interconnected matrices with appropriate dimensions. The initial state *κ*(*t*) is a continuously differentiable vector function. **U** is the bias value, and *τ*(*t*) denotes transmission delay and satisfies 0 ≤ *τ*(*t*) ≤ *τ*, *τ*′(*t*) ≤ *τ*^*∗*^ ≤ 1, where *τ* and *τ*^*∗*^ are constants.

In this paper, we define the norms of the *n* × *n* matrix **M**=(*a*_*ij*_)_*n*×*n*_ and the *n*-dimensional vector as follows:

(5) $$\begin{array}{c} \left\|M\right\|=\underset{1\leq j\leq n}{\max}\left\{\sum_{i=1}^{n}\left|a_{ij}\right|\right\},\\ \left\|x\left(t\right)\right\|=\sqrt{x{\left(t\right)}^{T}x\left(t\right)}. \end{array}$$

We assume that all activation functions *g*_*i*_(*i*=1,2,…, *n*) satisfy the following conditions:

- *g*_*i*_: **R**⟶**R** is continuous and differentiable, and *g*_*i*_(0)=0
- *g*_*i*_ is bounded on **R**, that is, ‖*g*_*i*_(*t*)‖ ≤ *G*_*i*_ for *t* ∈ **R**, and *G*_*i*_ is a constant
- ‖*g*_*i*_(*y*) − *g*_*i*_(*x*)‖‖≤*L*_*i*_‖‖*y* − *x*‖ for all *y*, *x* ∈ **R**, where *L*_*i*_ is a constant, and let *L*=max{*L*_1_, *L*_2_,…, *L*_*n*_}

**Lemma 1 .**

If ‖**A**^−1^(**B**+**C**)‖*L* < 1 and the activation function *g* satisfies conditions (i)–(iii), then the equilibrium point of system (4) must exist and be unique.

**Proof**

If **u**^*∗*^=(*u*_1_^*∗*^, *u*_2_^*∗*^,…,*u*_2_^*∗*^)^*T*^ is the equilibrium point of system (4), then

(6) $$\begin{array}{c} -Au^{*}+\left(B+C\right)g\left(u^{*}\right)+U=0. \end{array}$$

According to the definition of ***A***, the inverse matrix **A**^−1^ of ***A*** exists; therefore, (6) is equivalent to

(7) $$\begin{array}{c} -u^{*}+A^{-1}\left(B+C\right)g\left(u^{*}\right)+A^{-1}U=0. \end{array}$$

Let **W**=**A**^−1^(**B**+**C**)=(*w*_*ij*_)_*n*×*n*_ and **I**=**A**^−1^**U**, then (7) can be expressed as

(8) $$\begin{array}{c} -u^{*}+Wg\left(u^{*}\right)+I=0. \end{array}$$

To prove that (8) is true, we create the following mapping:

(9) $$\begin{array}{c} H\left(u\right)=Wg\left(u\right)+I. \end{array}$$

From conditions (i)–(iii), **g**(**u**) is a continuous mapping of **R**_*n*_⟶**R**_*n*_; then, ***H***(***u***) is also a continuous mapping of **R**_*n*_⟶**R**_*n*_. According to the definition of the norm of the *n*-dimensional vector and assumption (ii), we have that

(10) $$\begin{array}{c} \left\|H\right\|=\sqrt{\sum_{j=1}^{n}{\left(\sum_{i=1}^{n}\left(w_{ji}g_{i}\left(u_{i}\right)+I_{i}\right)\right)}^{2}}\\ \leq \sqrt{\sum_{j=1}^{n}{\left(\sum_{i=1}^{n}\left(\left|w_{ji}\right|G_{i}+\left|I_{i}\right|\right)\right)}^{2}}\\ \leq \sqrt{\sum_{j=1}^{n}{\left(\sum_{i=1}^{n}\left(\left|w_{ji}\right|G+\left|I_{i}\right|\right)\right)}^{2}}, \end{array}$$

where *G*=max{*G*_1_, *G*_2_,…, *G*_*n*_}.

Let $\rho \leq \sqrt{\sum_{j=1}^{n}{\left(\sum_{i=1}^{n}\left(\left|w_{ji}\right|G+\left|I_{i}\right|\right)\right)}^{2}}$, then Ω={**x***|*‖**x**‖ ≤ *ρ*} is a bounded convex set and ***H***(***u***) is a continuous mapping of Ω⟶Ω . According to Brouwer's fixed-point theorem, there must exist **u**^*∗*^ ∈ Ω such that **H**(**u**^*∗*^)=**u**^*∗*^. As formula (8) holds, there exists an equilibrium point **u**^*∗*^ in system (4). To prove the uniqueness of the equilibrium point, we suppose **v**^*∗*^=(*v*_1_^*∗*^, *v*_2_^*∗*^,…,*v*_*n*_^*∗*^)^*T*^ is another equilibrium point of system (4). Then,

(11) $$\begin{array}{c} \sum_{j=1}^{n}\left|u_{j}^{*}-v_{j}^{*}\right|=\sum_{j=1}^{n}\left|\sum_{i=1}^{n}w_{ji}\left(g_{i}\left(u_{i}^{*}\right)-g_{i}\left(v_{i}^{*}\right)\right)\right|\\ \leq \sum_{j=1}^{n}\left(\sum_{i=1}^{n}\left(\left|w_{ji}\right|\left|g_{i}\left(u_{i}^{*}\right)-g_{i}\left(v_{i}^{*}\right)\right|\right)\right)\\ =\sum_{i=1}^{n}\left(\sum_{j=1}^{n}\left(\left|w_{ji}\right|L_{i}\left|u_{i}^{*}-v_{i}^{*}\right|\right)\right)\\ \leq \sum_{i=1}^{n}\left(\left\|W\right\|L\left|u_{i}^{*}-v_{i}^{*}\right|\right)\\ \leq \left\|W\right\|L\sum_{i=1}^{n}\left|u_{i}^{*}-v_{i}^{*}\right|. \end{array}$$

We have

(12) $$\begin{array}{c} \sum_{j=1}^{n}\left|u_{j}^{*}-v_{j}^{*}\right|\left(1-\left\|W\right\|L\right)\leq 0, \end{array}$$

i.e.,

(13) $$\begin{array}{c} \sum_{j=1}^{n}\left|u_{j}^{*}-v_{j}^{*}\right|\left(1-\left\|A^{-1}\left(B+C\right)\right\|L\right)\leq 0. \end{array}$$

According to the condition 1 − ‖**A**^−1^(**B**+**C**)‖*L* > 0, we have ∑_*j*=1_^*n*^|*u*_*j*_^*∗*^ − *v*_*j*_^*∗*^|=0 and **v**^*∗*^=**u**^*∗*^. This equation shows that the equilibrium point is unique.

Let the equilibrium point of system (4) be **x**^*∗*^ and **y**(**t**)=**x**(**t**) − **x**^*∗*^. In this situation, system (4) can be rewritten as

(14) $$\begin{array}{c} \left\{\begin{array}{c} \dot{y}\left(t\right)=-Ay\left(t\right)+Bf\left(y\left(t\right)\right)+Cf\left(y\left(t-\tau \left(t\right)\right)\right),\\ y\left(t\right)=\eta \left(t\right),\;\forall t\in \left[-\tau ,0\right], \end{array}\right) \end{array}$$

where **f**(**y**)=**g**(**y**+**x**^*∗*^) − **g**(**x**^*∗*^), **f**(**y**(**t**))=(*f*_1_(*y*_1_(*t*)), *f*_2_(*y*_2_(*t*)),…,*f*_*n*_(*y*_*n*_(*t*))^*T*^, and the initial state is *η*(*t*)=**x**(*t*) − **x**^*∗*^, *t* ∈ [−*τ*, 0]. The meaning of the other symbols is the same as that of system (6). Let activation function *f*_*i*_(*z*)(*i*=1,2,…, *n*) be a continuous function that satisfies a Lipschitz condition for all *z* ∈ **R**. That is, assume that

(15) $$\begin{array}{c} \left|f_{i}\left(z_{1}\right)-f_{i}\left(z_{2}\right)\right|\leq L_{i}\left|z_{1}-z_{2}\right|, \end{array}$$

for some constant *L*_*i*_ > 0 and for all *z*_1_, *z*_2_ ∈ **R**.

**Definition 1 .**

System (14) is said to be globally exponentially stable, if there exists a constant **M** ≥ 1 and *α* > 0, such that

(16) $$\begin{array}{c} \left\|y\left(t\right)\right\|\leq M\underset{s\in \left[-\tau ,0\right]}{\sup}\left(\left\|y\left(s\right)\right\|\right)\exp\left(-\alpha t\right),\;\forall t>0. \end{array}$$

**Lemma 2 (Gronwall's inequality [31]).**

Let *K* be a nonnegative constant and *v*(*t*) and *p*(t) are nonnegative and continuous functions on the interval *α* ≤ *t* ≤ *β* and satisfy the inequality

(17) $$\begin{array}{c} v\left(t\right)\leq K+\int_{a}^{b}p\left(s\right)v\left(s\right)\text{d}s,\;a\leq t\leq b, \end{array}$$

then

(18) $$\begin{array}{c} v\left(t\right)\leq K\;\exp\left(\int_{a}^{b}p\left(s\right)\text{d}s\right),\;a\leq t\leq b. \end{array}$$

## 2. Stability Analysis

In this section, we discuss the global exponential stability condition for the trivial solution of system (14).

The linear term in system (14) can be expressed as

(19) $$\begin{array}{c} \dot{y}\left(t\right)=-Ay\left(t\right). \end{array}$$

The fundamental solution matrix of (19) is

(20) $$\begin{array}{c} \exp\left[-At\right]=\left[\begin{array}{cccc} e^{-a_{1}t} & \; & \; & 0\\ \; & e^{-a_{2}t} & \; & \;\\ \; & \; & \ddots & \;\\ 0 & \; & \; & e^{-a_{n}t} \end{array}\right]. \end{array}$$

Let the initial time *t*=0 and the corresponding initial value be *η*(0)=(*η*_1_, *η*_2_,…,*η*_*n*_)^*T*^, then the solution of system (19) can be expressed as

(21) $$\begin{array}{c} Y\left(t\right)=\exp\left(-At\right)\eta \left(0\right). \end{array}$$

For convenience, we denote *ω*=min{*a*_1_, *a*_2_,…, *a*_*n*_}.

**Theorem 1 .**

Suppose that the activation function **f**(•) satisfies conditions (i)–(iii) with the Lipschitz constant *L*; if

(22) $$\begin{array}{c} \left(\left\|B\right\|+\left\|C\right\|\right)L\frac{1}{1-\tau^{*}}\exp\left(\omega \tau \right)-\omega <0, \end{array}$$

then the trivial solution of system (14) is globally exponentially stable.

**Proof**

For *t*=0, the initial value is *η*=(*η*_1_, *η*_2_,…,*η*_*n*_)^*T*^; by using the method of constant variation, we obtain that the solution of system (14) satisfies the following equation:

(23) $$\begin{array}{c} y\left(t\right)=\exp\left(-At\right)\eta +\int_{0}^{t}\exp\left(-A\left(t-s\right)\right)\left(Bf\left(y\left(s\right)\right)+Cf\left(y\left(s-d\left(s\right)\right)\right)\right)\text{d}s. \end{array}$$

Taking the norm on both sides of the above formula, without loss of generality, for *t* > *τ*, we obtain

(24) $$\begin{array}{c} \left\|y\left(t\right)\right\|\leq \left\|\exp\left(-At\right)\eta \right\|+\left\|\int_{0}^{t}\exp\left(-A\left(t-s\right)\right)\left(Bf\left(y\left(s\right)\right)+Cf\left(y\left(s-d\left(s\right)\right)\right)\right)\text{d}s\right\|\\ \leq \left\|\exp\left(-At\right)\eta \right\|+\left\|\int_{0}^{t}\exp\left(-A\left(t-s\right)\right)Bf\left(y\left(s\right)\right)\text{d}s\right\|\\ +\left\|\int_{0}^{t}\exp\left(-A\left(t-s\right)\right)Cf\left(y\left(s-\tau \left(s\right)\right)\right)\text{d}s\right\|\\ \leq \;\exp\left(-\omega t\right)\left\|\eta \right\|+\int_{0}^{t}\left\|\exp\left(-A\left(t-s\right)\right)\right\|\left\|B\right\|\left\|f\left(y\left(s\right)\right)\right\|\text{d}s\\ +\int_{0}^{t}\left\|\exp\left(-A\left(t-s\right)\right)\right\|\left\|C\right\|\left\|f\left(y\left(s-\tau \left(s\right)\right)\right)\right\|\text{d}s\\ \leq \;\exp\left(-\omega t\right)\left\|\eta \right\|+\int_{0}^{t}\left\|\exp\left(-\omega \left(t-s\right)\right)\right\|\left\|B\right\|L\left\|y\left(s\right)\right\|\text{d}s\\ +\int_{0}^{t}\exp\left(-\omega \left(t-s\right)\right)\left\|C\right\|L\left\|y\left(s-\tau \left(s\right)\right)\right\|\text{d}s\;\\ \leq \;\exp\left(-\omega t\right)\left\|\eta \right\|+\int_{0}^{t}\left\|\exp\left(-\omega \left(t-s\right)\right)\right\|\left\|B\right\|L\left\|y\left(s\right)\right\|\text{d}s\\ +\int_{0}^{t}\exp\left(-\omega \left(t-s\right)\right)\left\|C\right\|L\left\|y\left(s-\tau \left(s\right)\right)\right\|\frac{1}{1-\tau P\left(s\right)}\text{d}\left(s-\tau \left(s\right)\right)\;\\ \leq \;\exp\left(-\omega t\right)\left\|\eta \right\|+\int_{0}^{t}\left\|\exp\left(-\omega \left(t-s\right)\right)\right\|\left\|B\right\|L\left\|y\left(s\right)\right\|\text{d}s\;\\ +\int_{-\tau \left(s\right)}^{t-\tau \left(s\right)}\exp\left(-\omega \left(t-s-\tau \left(s\right)\right)\right)\left\|C\right\|L\left\|y\left(s\right)\right\|\frac{1}{1-\tau^{*}}\text{d}s\\ \leq \;\exp\left(-\omega t\right)\left\|\eta \right\|+\int_{0}^{t}\left\|\exp\left(-\omega \left(t-s\right)\right)\right\|\left\|B\right\|L\left\|y\left(s\right)\right\|\text{d}s\\ +\int_{-\tau }^{t}\exp\left(-\omega \left(t-s-\tau \right)\right)\left\|C\right\|L\left\|y\left(s\right)\right\|\frac{1}{1-\tau^{*}}\text{d}s\\ \leq \;\exp\left(-\omega t\right)\left\|\eta \right\|+\int_{0}^{t}\left\|\exp\left(-\omega \left(t-s\right)\right)\right\|\left\|B\right\|L\left\|y\left(s\right)\right\|\text{d}s,\\ +\int_{-\tau }^{t}\exp\left(-\omega \left(t-s-\tau \right)\right)\left\|C\right\|L\left\|y\left(s\right)\right\|\frac{1}{1-\tau^{*}}\text{d}s\\ \leq \;\exp\left(-\omega t\right)\left\|\eta \right\|+\left\|B\right\|L\;\exp\left(-\omega t\right)\int_{-\tau }^{t}\exp\left(\omega s\right)\left\|y\left(s\right)\right\|\text{d}s\\ +\left\|C\right\|L\frac{1}{1-\tau^{*}}\exp\left(-\omega t\right)\exp\left(\omega \tau \right)\int_{-\tau }^{t}\exp\left(\omega s\right)\left\|y\left(s\right)\right\|\text{d}s\\ =\exp\left(-\omega t\right)\left\|\eta \right\|\\ +\left(\left(\left\|B\right\|+\left\|C\right\|\right)L\frac{1}{1-\tau^{*}}\exp\left(\omega \tau \right)\right)\exp\left(-\omega t\right)\int_{-\tau }^{t}\exp\left(\omega s\right)\left\|y\left(s\right)\right\|\text{d}s. \end{array}$$

According to Lemma 1 (Gronwall's inequality), we obtain

(25) $$\begin{array}{c} \exp\left(\omega t\right)\left\|y\left(t\right)\right\|\leq \left\|\eta \right\|\exp\left(\left(\left\|B\right\|L+\left\|C\right\|L\frac{1}{1-\tau^{*}}\exp\left(\omega \tau \right)\right)\int_{-\tau }^{t}\text{d}s\right)\\ =\left\|\eta \right\|\exp\left(\left(\left(\left\|B\right\|+\left\|C\right\|\right)L\frac{1}{1-\tau^{*}}\exp\left(\omega \tau \right)\right)\left(t+\tau \right)\right)\\ =\left\|\eta \right\|\exp\left(\left(\left(\left\|B\right\|+\left\|C\right\|\right)L\frac{1}{1-\tau^{*}}\exp\left(\omega \tau \right)\right)\tau \right)\\ \cdot \;\exp\left(\left(\left(\left\|B\right\|+\left\|C\right\|\right)L\frac{1}{1-\tau^{*}}\exp\left(\omega \tau \right)\right)t\right). \end{array}$$

Therefore,

(26) $$\begin{array}{c} \left\|y\left(t\right)\right\|\leq \left\|\eta \right\|\exp\left(\left(\left(\left\|B\right\|+\left\|C\right\|\right)L\frac{1}{1-\tau^{*}}\exp\left(\omega \tau \right)\right)\tau \right)\\ \cdot \;\exp\left(\left(\left(\left\|B\right\|+\left\|C\right\|\right)L\frac{1}{1-\tau^{*}}\exp\left(\omega \tau \right)\right)-\omega \right)t. \end{array}$$

Since ((‖**B**‖+‖**C**‖)*L*(1/1 − *τ*^*∗*^)exp(*ωτ*)) − *ω* < 0, then the delay system (14) is globally exponentially stable. The proof is completed.

**Remark 1 .**

How to obtain better stability results in time-delay systems has been the concern of many scholars. Some scholars use improved integral inequality techniques and construct better Lyapunov–Krasovskii functional and estimate its derivative to obtain new results. In [29], the authors discuss the exponential stability and generalized dissipative analysis of time-delay generalized neural networks. Based on Lyapunov–Krasovskii functional (LKF) and Wirtinger single integral inequality (WSII) and Wirtinger double integral inequality (WDII) techniques, they establish new criteria for exponential stability of generalized neural networks with delays. However, as we see, their results are still based on the assumption that there are some unknown symmetric matrices. They only used some examples to verify the validity of the results, but failed to prove the existence of these unknown symmetric matrices theoretically. In this paper, the stability criterion is only related to the coefficient matrix of the system and has nothing to do with other unknown matrices.

Next, we consider several special cases.

For the following system without time delay,

(27) $$\begin{array}{c} \left\{\begin{array}{c} \dot{y}\left(t\right)=-Ay\left(t\right)+Bf\left(y\left(t\right)\right),\\ y\left(0\right)=\eta \left(0\right), \end{array}\right) \end{array}$$

we have the following corollary.

**Corollary 1 .**

Suppose that the activation function satisfies the Lipschitz condition; if ‖**B**‖*L* − *ω* < 0, the trivial solution of system (27) is globally exponentially stable.

For the following system with constant time delay,

(28) $$\begin{array}{c} \left\{\begin{array}{c} \dot{x}\left(t\right)=-Ax\left(t\right)+Bg\left(x\left(t\right)\right)+Cg\left(x\left(t-\tau \right)\right)+U,\\ x\left(t\right)=\kappa \left(t\right),\;\forall t\in \left[-\tau ,0\right], \end{array}\right) \end{array}$$

we can get the following corollary.

**Corollary 2 .**

Suppose that the activation function satisfies the Lipschitz condition; if ((‖**B**‖+‖**C**‖)*L*  exp(*ωτ*)) − *ω* < 0, the trivial solution of system (28) is globally exponentially stable.

## 3. Numerical Examples

In this section, we provide four illustrative examples to demonstrate the effectiveness of Theorem 1.

**Example 1 .**

We consider the following two-dimensional neural network model without delay:

(29) $$\begin{array}{c} \left(\begin{array}{c} \dot{x}\left(t\right)\\ \dot{y}\left(t\right) \end{array}\right)=-A\left(\begin{array}{c} x\left(t\right)\\ y\left(t\right) \end{array}\right)+B\left(\begin{array}{c} f_{1}\left(x\left(t\right)\right)\\ f_{2}\left(y\left(t\right)\right) \end{array}\right), \end{array}$$

where the activation function*f*_*i*_(*u*)=tanh(*u*)(*i*=1,2) satisfies the Lipschitz condition with the Lipschitz constant *L*=1.

**Figure 1:**
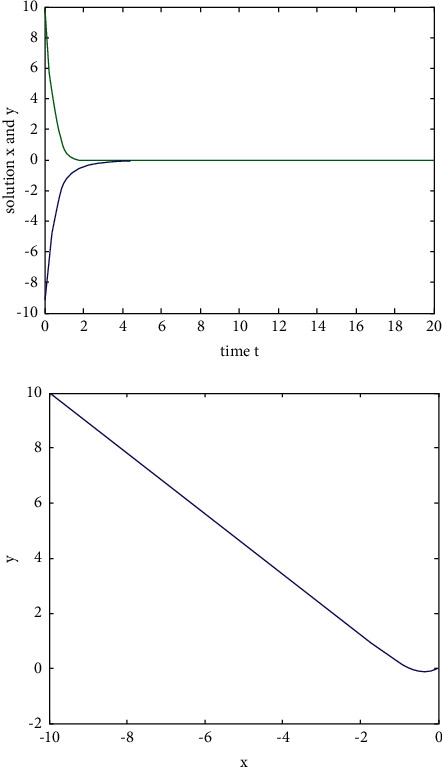
The state rail diagram of system (29): (*L*‖**B**‖ − *ω* < 0).

We take

(30) $$\begin{array}{c} A=\left[\begin{array}{cc} 2 & 0\\ 0 & 2 \end{array}\right],\\ B=\left[\begin{array}{cc} 1 & 0.8\\ 0.4 & -0.5 \end{array}\right], \end{array}$$

and then, for *ω*=2, ‖**B**‖=1.4 and *L*‖**B**‖ − *ω*=−0.6 < 0. When *t*=0, the initial value is (*x*(0), *y*(0))=(−10,10). According to Corollary 1, the zero solution of system (31) is exponentially stable, and the state rail diagram of the system is shown in Figure 1.

**Figure 2:**
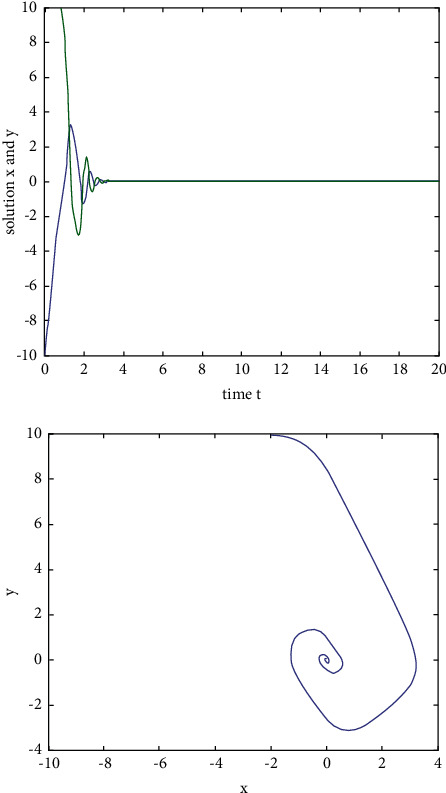
The state rail diagram of system (29): (*L*‖**B**‖ − *ω* < 0).

If we take $A=\left[\begin{array}{cc} 2 & 0\\ 0 & 1 \end{array}\right]$ and $B=\left[\begin{array}{cc} 5 & -20\\ 10 & -10 \end{array}\right]$, then *ω*=1, ‖**B**‖=30, and *L*‖**B**‖ − *ω*=28 > 0. When *t*=0, the initial value is (*x*(0), *y*(0))=(−10,10). The state rail diagram of the system is shown in Figure 2. According to the literature [36], we know that system (31) is global asymptotic stability, but we can see that it is not exponentially stable from Figure 2.

**Example 2 .**

We consider the following two-dimensional neural network model with constant delay:

(31) $$\begin{array}{c} \left(\begin{array}{c} \dot{x}\left(t\right)\\ \dot{y}\left(t\right) \end{array}\right)=-A\left(\begin{array}{c} x\left(t\right)\\ y\left(t\right) \end{array}\right)+B\left(\begin{array}{c} f_{1}\left(x\left(t\right)\right)\\ f_{2}\left(y\left(t\right)\right) \end{array}\right)+C\left(\begin{array}{c} f_{1}\left(x\left(t-\tau \right)\right)\\ f_{2}\left(y\left(t-\tau \right)\right) \end{array}\right), \end{array}$$

where the activation function *f*_*i*_(*u*)=tanh(*u*), *i*=1,2, satisfies the Lipschitz condition and *L*=1.

**Figure 3:**
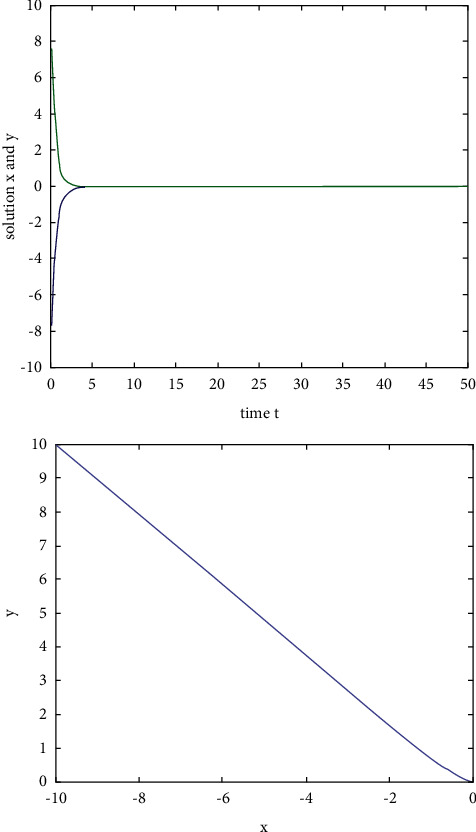
The state rail diagram of system (31): ((‖**B**‖+‖**C**‖)*L*  exp(*ωτ*) − *ω* < 0).

If we take

(32) $$\begin{array}{c} A=\left[\begin{array}{cc} 2 & 0\\ 0 & 2 \end{array}\right],\\ B=\left[\begin{array}{cc} 0.1 & 0.3\\ 0.2 & -0.2 \end{array}\right],\\ C=\left[\begin{array}{cc} 0.2 & -0.1\\ -0.4 & 0.3 \end{array}\right], \end{array}$$

time delay *τ*=0.1, *ω*=2, ‖**B**‖=0.8, and ‖**C**‖=1, then ((‖**B**‖+‖**C**‖)*L*  exp(*ωτ*)) − *ω* ≈ −0.0107 < 0. According to Corollary 2, the zero solution of system (33) is exponentially stable. When *t*=0, the initial value is (*x*(0), *y*(0))=(−10,10). The state rail diagram of the system is shown in Figure 3.

**Example 3 .**

We consider the following two-dimensional neural network model with variable delay:

(33) $$\begin{array}{c} \left(\begin{array}{c} \dot{x}\left(t\right)\\ \dot{y}\left(t\right) \end{array}\right)=-A\left(\begin{array}{c} x\left(t\right)\\ y\left(t\right) \end{array}\right)+B\left(\begin{array}{c} f_{1}\left(x\left(t\right)\right)\\ f_{2}\left(y\left(t\right)\right) \end{array}\right)+C\left(\begin{array}{c} f_{1}\left(x\left(t-\tau \left(t\right)\right)\right)\\ f_{2}\left(y\left(t-\tau \left(t\right)\right)\right) \end{array}\right), \end{array}$$

where the activation function *f*_*i*_(*u*)=tanh(*u*), *i*=1,2, satisfies the Lipschitz condition and*L*=1.

**Figure 4:**
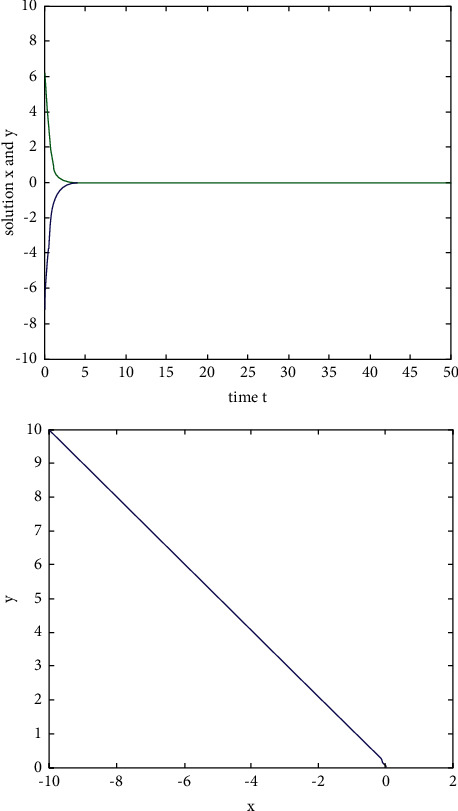
The state rail diagram of system (33): ((‖**B**‖+‖**C**‖)*L*  exp(*ωτ*)/(1 − *τ*^*∗*^) − *ω* < 0).

If we take

(34) $$\begin{array}{c} A=\left[\begin{array}{cc} 2 & 0\\ 0 & 2 \end{array}\right],\\ B=\left[\begin{array}{cc} 0.1 & 0.3\\ 0.2 & -0.2 \end{array}\right],\\ C=\left[\begin{array}{cc} 0.2 & -0.1\\ -0.4 & 0.3 \end{array}\right], \end{array}$$

and time delay *τ*(*t*)=0.1  sin(*t*)+0.1, then *ω*=2, ‖**B**‖=0.5, ‖**C**‖=0.6, *τ*=0.2,  *τ*^*∗*^=0.1, and ((‖**B**‖+‖**C**‖)*L*(1/1 − *τ*^*∗*^)exp(*ωτ*)) − *ω* ≈ −0.1767 < 0. According to Theorem 1, the zero solution of system (33) is exponentially stable. When *t*=0, the initial value is(*x*(0), *y*(0))=(−10,10), and the state rail diagram of the system is shown in Figure 4.

**Figure 5:**
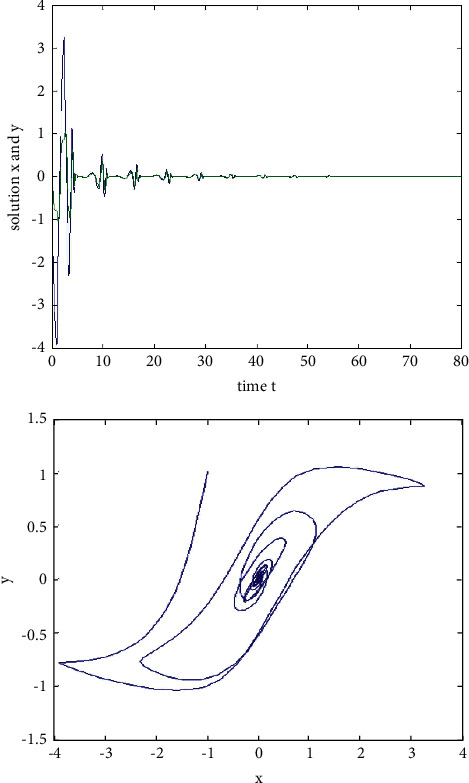
The state rail diagram of system (33): ((‖**B**‖+‖**C**‖)*L*  exp(*ωτ*)/(1 − *τ*^*∗*^) − *ω* < 0).

If we take

(35) $$\begin{array}{c} A=\left[\begin{array}{cc} 2 & 0\\ 0 & 2 \end{array}\right],\\ B=\left[\begin{array}{cc} 0.1 & 0.3\\ 0.2 & -0.2 \end{array}\right],\\ C=\left[\begin{array}{cc} 0.2 & -0.1\\ -0.4 & 0.3 \end{array}\right], \end{array}$$

and time delay *τ*(*t*)=0.1  sin(*t*)+0.1, then *ω*=2, ‖**B**‖=11, ‖**C**‖=10.3, *τ*=0.2, *τ*^*∗*^=0.1,  and ((‖**B**‖+‖**C**‖)*L*(1/1 − *τ*^*∗*^)exp(*ωτ*)) − *ω* ≈ 33.31 > 0. When *t*=0, the initial value is (*x*(0), *y*(0))=(−1,1). The state rail diagram of the system is shown in Figure 5. According to the literature [36], we know that system (33) is global asymptotic stability, but we can see that it is not exponentially stable from Figure 5.

**Remark 2 .**

In [30], the author also gives a two-dimensional example. According to the criterion of exponential stability obtained in paper [30], it is necessary to find some symmetric matrices that meet the specified matrix inequalities. Although the authors can find these matrices, the results obtained by this method are accidental and uncertain and they cannot guarantee the existence of symmetric matrices that meet the conditions. The stability judgment method used in the example in this paper is according to the data of the coefficient matrix without the unknown parameters or matrix of the third party. Although the result is relatively conservative, this is a sufficient condition and has obvious advantages for judging the stability of the system.

## 4. Conclusion

In this work, we have studied the exponential stability for the Hopfield neural network with a time-varying delay. We use the method of variation of constants of ordinary differential equations to obtain an equation satisfied by the state variable of the neural network. Then, we used Gronwall's inequality to analyze this system and obtained new criteria for the exponential stability of the neural networks with time-varying delay. Our result is related only to the coefficient matrix of the system and not to the existence of the other unknown matrices. It is easy to test the exponential stability for specific systems by using these criteria.

## Data Availability

The data used to support the findings of this study are included within the article.
